# Temporomandibular disorders. A case-control study

**DOI:** 10.4317/medoral.18040

**Published:** 2012-05-01

**Authors:** Rafael Poveda-Roda, Jose V. Bagán, Jose M. Sanchis, Enrique Carbonell

**Affiliations:** 1 MD PhD. Assistant in Stomatology. Departament of Stomatology and Maxillofacial Surgery. General Universitary Hospital of Valencia; 2 MD PhD. Professor and Chairman. Departament of Stomatology and Maxillofacial Surgery. General Universitary Hospital of Valencia

## Abstract

Objective: To compare the risk factors and clinical manifestations of patients with temporomandibular disorders (TMDs) diagnosed according to the Research Diagnostic Criteria for Temporomandibular Disorders (RDC/TMD) (axis I) versus an age and gender matched control group. Study Design: A total of 162 patients explored according to the RDC/TMD (mean age 40.6±18.8 years, range 7-90; 11.1% males and 88.9% females) were compared with 119 controls, measuring differences in TMD risk factors (sleep disturbances, stress, psychoactive medication, parafunctions, loss of posterior support, ligament hyperlaxity) and clinical variables (joint sounds, painful muscle and joint palpation, maximum aperture). Results: Myofascial pain (MFP) (single or multiple diagnoses) was the most frequent diagnosis (42%). The most common diagnostic combination was MFP plus arthralgia (16.0%). Statistically significant differences were observed in clenching (OR 2.3; 95%CI: 1.4-3.8) and in maximum active aperture (MAA) on comparing the two groups both globally (TMD vs. controls) (patients 36.7±8.6 mm, controls 43.1±5.8 mm; F=45.41, p = 0.000) and on comparing according to diagnostic categories. MFP explained most of the observed differences in the risk factors: stress perception (OR=1.98;I.C.:1.01-3.89), psychoactive medication (OR=2.21; I.C.:1.12-4.37), parafunctions (OR=2.14;I.C.:1.12-4.11), and ligament laxity (OR=2.6;I.C.:1.01-6.68). Joint sounds were more frequent in patients with MFP (39.7% vs. 24.0%; χ2=4.66; p=0.03), and painful joint palpation was more common in patients with disc displacement with reduction (DDWR)(15.9% vs. 5.0%; χ2 = 5.2; p = 0.02) and osteoarthrosis (20.8% vs. 5.0%; χ2 = 7.0; p = 0.008). Conclusions: There is a high prevalence of signs and symptoms of TMDs in the general population. Significant differences are observed in clenching and MAA between patients and controls considered both globally and for each diagnostic category individually. The analyzed risk factors (except loss of posterior support) show a statistically significant OR for the diagnosis of MFP.

** Key words:**Disorder, dysfunction, temporomandibular, myofascial, osteoarthrosis, TMJ, disc displacement.

## Introduction

Temporomandibular disorders (TMDs) are essentially characterized by the appearance of pain, joint sounds and alterations in mandibular movement. Affected patients may experience one or more of these manifestations simultaneously, and their prevalence is relatively high in the general population (1). In many cases the disorders are not perceived as affecting quality of life; as a result, the proportion of patients seeking medical help for TMDs is limited: between 6-7% (2) and 8.4% (3). The classification of TMDs is also controversial. A diagnostic model was proposed in 1992, known as the Research Diagnostic Criteria for Temporomandibular Disorders (RDC/TMD) (4). Since its publication, the RDC/TMD has been widely used in epidemiological, clinical and experimental studies. The RDC/TMD is based on a series of protocolized clinical procedures and on strict diagnostic criteria applied to the most common types of TMD (5). Two diagnostic axes are contemplated: axis I establishes a diagnosis based on clinical variables, while axis II establishes a diagnosis based on psychological variables. Three major diagnostic categories are contemplated in axis I (myofascial pain, disc alterations and arthralgia-arthritis-arthrosis), each with several subcategories. The RDC/TMD is presently undergoing revision in an attempt to improve certain aspects such as the diagnostic algorithm of myofascial pain and disc displacement without reduction, its accuracy and its suitability for clinical use (6). The risk factors for TMD have also been the subject of numerous studies. The factors that have centered most attention are patient gender, stress, parafunctional habits, occlusal factors and ligament hyperlaxity (7). However, to date there have been few publications on this subject in the Spanish language literature. In conducting a search in Medline with the term RDC/TMD we did not find any article of Spanish or Latin American author on the diagnosis and risk factors of TMD with a case-control design.

The present study compares the risk factors and clinical manifestations of patients with TMJDs diagnosed according to the RDC/TMD axis I criteria versus an age- and gender-matched control group.

## Material and Methods

The study was approved by the Clinical Research Ethics Committee of Valencia University General Hospital (Valencia, Spain).

Between September 2008 and November 2009 we explored 202 patients referred to the Department of Stomatology of the Hospital due to TMDs. Exploration was carried out according to the RDC/TMD axis I criteria, in accordance with routine clinical practice. A total of 162 patients were diagnosed with one or more of the categories included in the RDC/TMD. Written informed consent for data inclusion in the study was obtained from these subjects. Information was collected in relation to the risk factors for TMDs: sleep disturbances, stress perception, regular consumption of psychoactive medication (particularly antidepressants, anxiolytics, sleep inducers), parafunctional habits, loss of posterior occlusal support and ligament hyperlaxity as determined by the Beighton test (positivity being taken as a score of ≥ 4).

The diagnostic category disc displacement without reduction (DDWoR) was not included in the analysis.

The control group consisted of 119 subjects age- and gender-matched to the study group and selected from among the patients referred to the Department of Stomatology due to reasons other than TMD. Exclusion criteria in the control group were subjects with a history of malignant disease or major surgery in the orofacial region, minor surgery in the previous three months, cervico-facial chemo- or radiotherapy, and recovery from facial injuries.

All data were analyzed using the SPSS version 15.0 statistical package (Chicago, IL, USA). Dichotomic variables were compared using the chi-squared test. In the case of variables showing statistically significant differences, the latter were quantified based on the odds ratio (OR) and corresponding 95% confidence interval. Quantitative variables in turn were compared using ANOVA. Receiver Operating Characteristic (ROC) curves were used to assess the diagnostic yield of maximum interincisal aperture in the diagnosis of TMDs and their categories. In all cases, statistical significance was accepted for p<0.05.

## Results

**Figure 1:**
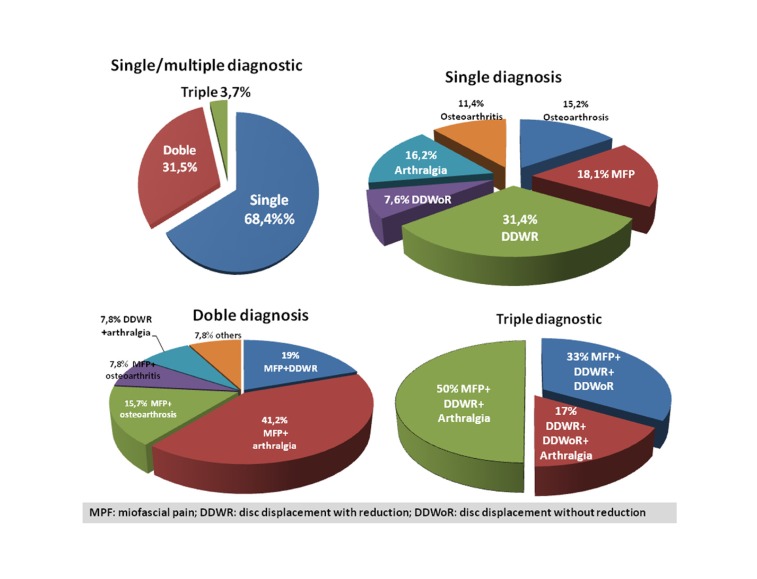
Diagnoses of the studied patients.

The mean age of the study group was 40.6 ±18.8 years (range 7-90 years); 11.1% were males and 88.9% females. The mean age in the control group was 40.5±19.9 years (13.4% males). There were no significant differences in gender (Pearson chi-squared test; χ2 = 0.35; p = 0.55) or age between the two groups (ANOVA; F = 0.024; p = 0.88). The Snedecor test showed the variances to be homogeneous (Fs = 1.11; p > 0.05). The study variables (demographic, clinical, risk factors) are shown in ( Table 1). (Fig. 1) describes the diagnoses of the patients in the study group. Myofascial pain (MFP) (single or multiple diagnosis) was the most frequent diagnosis (n = 68; 42%), followed by disc displacement with reduction (DDWR) (32.1%), arthralgia (30%), osteoarthrosis (14.2%), osteoarthritis (12.3%) and disc displacement without reduction (DDWoR) (8.6%). More than one RDC/TMD diagnosis was established in 35.2% of the patients. The most common diagnostic combination was MFP plus arthralgia (n = 26; 16.0%). Five patients presented three diagnoses each. In those patients with more than one diagnosis, the most frequent disorder was MFP (84.3% of the patients with 2 diagnosis, and 83.3% of the patients with 3 diagnoses).

**Table 1:**
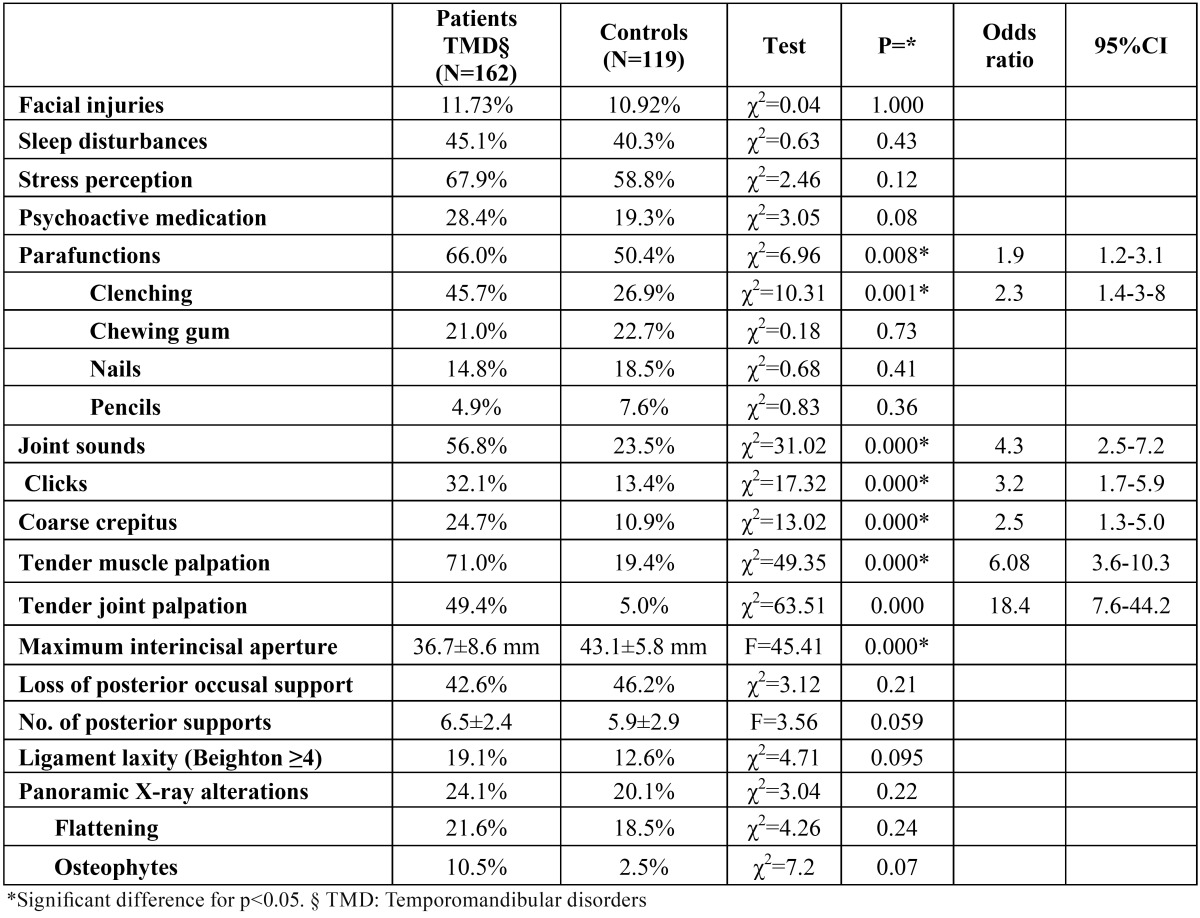
Global differences in risk factors and clinical variables between the groups.

On globally comparing the patients and controls ( Table 1), significant differences were only observed in the overall frequency of parafunctions -specifically clenching (OR 2.3; 95%CI 1.4-3.8).

In the control group, the incidence of signs of TMD varied between the 5% for painful joint palpation and the 23% for the presence of joint sounds.

**Figure 2:**
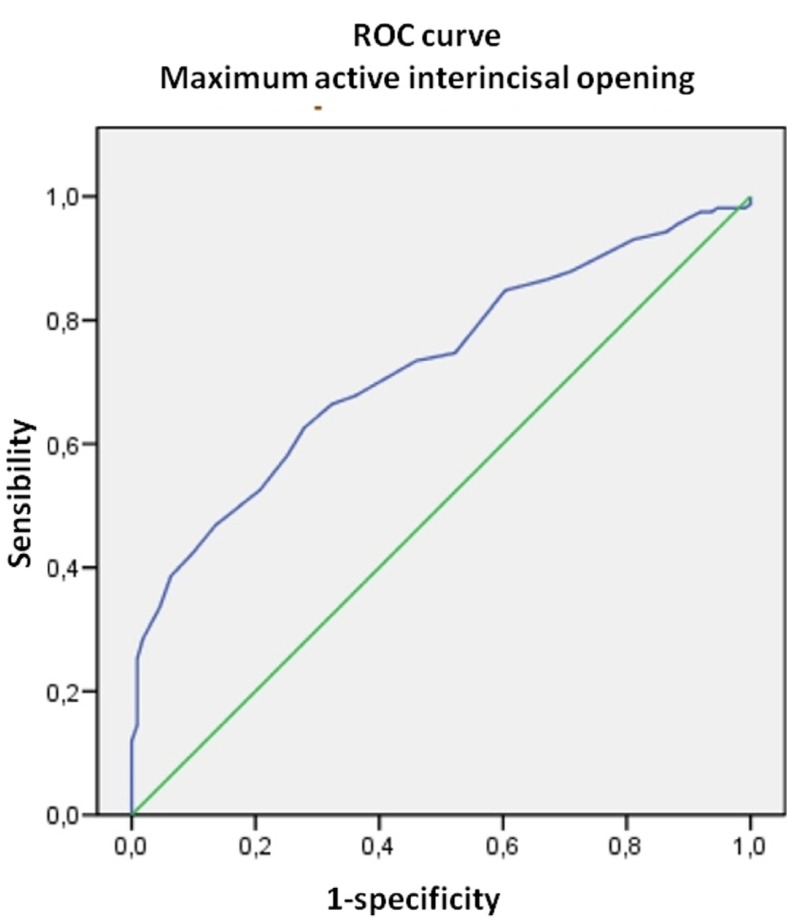
ROC curve. Diagnostic yield of maximum interincisal aperture.

Maximum active aperture (MAA) (the distance between the incisal margins of the upper and lower central incisors on instructing the subject to open the mouth as much as possible) was significantly greater in the controls both on comparing with the patients globally and with the patients according to the different diagnostic categories (MFP, DDWR, arthralgia and osteoarthritis/osteoarthrosis) ( Table 2). Considering these differences, ROC curves were plotted to determine whether MAA could be of diagnostic usefulness in discriminating patients with TMD. The area under the curve (AUC) was 72.6% (CI 66.7-78.5) (Fig. 2), the optimum cutoff point being 34.5 mm, with a sensitivity of 62.7% and a specificity of 72.1%. On performing the same procedure for each of the diagnostic categories, the results obtained were very similar to those of the global patient group, except for the category arthritis/arthrosis, where the optimum cutoff point was 32.5 mm, with a sensitivity of 33.3% and a specificity of 96.6%.

**Table 2:**
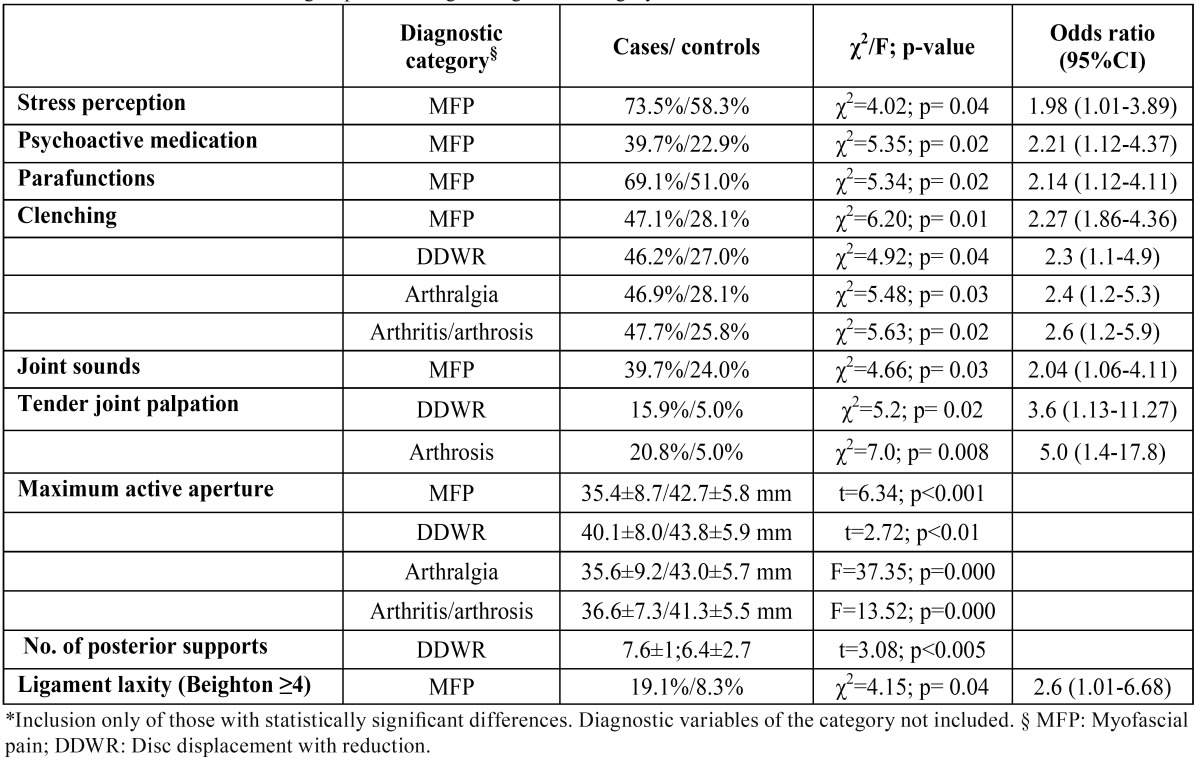
Differences between the groups according to diagnostic category.

In the analysis of risk factors according to diagnostic categories ( Table 2), MFP accounted for most of the differences observed in ( Table 1) (stress perception, psychoactive medication, global parafunctions, and ligament laxity). Clenching was significantly more frequent in all the diagnostic categories than in the respective controls. The clinical variables included in ( Table 2) (joint sounds and tender joint palpation) were only analyzed in those categories in which they did not represent a diagnostic criterion. In this context, joint sounds were significantly more frequent in patients with MFP, and painful joint palpation was significantly more common in the patients DDWR and arthrosis.

## Discussion

In our study we selected a control group from among the patients seeking dental care instead of a general population sample, in order to control potential bias associated with the variable “active seeking of treatment”. Although signs and symptoms of TMD are frequent in the general population, people are considerably less likely to seek treatment probably because such disorders are considered to have only a slight or moderate impact upon quality of life, and in many cases tend to improve or resolve spontaneously (8).

Disc displacement without reduction (DDWoR) was not included in the comparison according to diagnostic categories because of its low prevalence and the limited validity and precision of the RDC/TMD in this process (6).

Approximately two-thirds of our patients presented a single diagnosis –the most common being DDWR (31.4%), followed by MFP (18.1%). These figures differ from those recorded by our group in an earlier study (9) involving a series of 850 patients different from those of the present study but pertaining to the same geographical and social setting (DDWR 44.8% and MFP 35.2%). On the other hand, the present study registered a considerable increase in the proportion of patients diagnosed with osteoarthritis/osteoarthrosis (from 13.4% in the previous study to 27.2% in the present series).

Parafunctions, and specifically patient perceived clenching, were the only risk factor establishing significant differences between patients with TMD and the controls. Melis et al. (10) recorded clenching in 27.2% of the general population –this percentage being very similar to our own figure (26.9%) in the control group. Huang et al. (11) in turn found clenching to be associated with a diagnosis of MFP (OR = 4.8) and MFP plus arthralgia (OR = 3.3). These results partly coincide with our own, with slightly lower ORs (2.27 and 2.4, respectively). In coincidence with Carlsson et al. (12), it can be concluded that clenching is positively correlated to the treatment demand for TMD. While other parafunctions are often cited as risk factors for TMD, and in some cases have been related to the appearance of temporomandibular joint pain (13), we observed no differences between our cases and controls, and no studies involving a design similar to our own have been found to report such differences.

Stress perception and psychoactive drug consumption did not establish differences between the cases and controls when compared globally, though significant differences were recorded in the diagnostic category MFP. This observation partly coincides with the data of an earlier study comparing a group of patients with MFP and a group of patients with joint disease versus a control group. The two study groups showed greater distress than the control group (14). In line with our results, Huang et al. (11) reported a significant association between somatization and MFP (with or without arthralgia) (OR 3.7-5.1), but not between somatization and arthralgia alone.

In 2003, Sarita et al. (15) reported that the partial or total loss of posterior occlusal support was not associated to the development of signs of TMJD in a group of subjects in Tanzania. We included this variable in our study and found no differences between cases and controls; indeed, the number of contacts was seen to be greater among the patients than in the controls –the difference reaching statistical significance in the case of the diagnostic category DDWR. It must be pointed out that the method used to select the control group in our study may have introduced bias in the form of the inclusion of a high proportion of patients with dental problems that may have required tooth extraction. Nevertheless, based on the results obtained, the loss of posterior support was not identified as a risk factor for TMD.

The use of psychoactive medication (p=0.08) and ligament laxity (p=0.09) showed no statistically significant differences between patients and controls, though the observed tendency could be of clinical relevance. In patients with syndromic ligament hyperlaxity (Marfan syndrome and Ehler-Danlos disease), joint sounds and temporomandibular joint dislocations are significantly more frequent than in the normal population (16).

In contrast to the above, maximum active aperture (MAA) active did show differences between the patients and controls both globally and in relation to each of the diagnostic categories. The RDC/TMD criteria establish limits for DDWoR with limitation of aperture (35 mm) and for MFP with limitation of aperture (40 mm). The mean MAA values recorded in our study for the normal population (control group) are far from the 53-58 mm that Agerberg (17) regards as normal, and are consistent with the 41.1±3.5 mm reported by Fukui et al. (18) for a young adult population without TMD. On exploring an optimum cutoff point for differentiating between patients and controls, we calculated a value of about 35 mm, except in the case of arthritis/arthrosis, where the cutoff point was in the order of 32 mm. The sensitivity and specificity values of these cutoff points were clearly less than acceptable; as a result, maximum aperture alone is not indicative of temporomandibular joint morbidity, except in extreme cases.

The recorded clinical manifestations logically reflect very significant differences between the cases and controls. From our point of view, the relevant finding is the high percentage of individuals in the control group presenting one or more manifestations (23.5% showed joint sounds, 19.4% had one or more painful masticatory muscle points, and 5% reported pain in response to palpation of the lateral or posterior pole of one or both temporomandibular joints), and which in global terms coincides with the data of other authors (19). Similar findings are observed related to the alterations identified on the panoramic X-rays, where 20.1% of the controls presented some type of alteration. The most frequent anomaly was condylar flattening, which moreover poses serious diagnostic reproducibility problems, as evidenced by Crow et al. (20) - with an inter-examiner Kappa index of 0.29. These authors observed some type of condylar change in 87% of the controls, with no differences between the latter and the group of patients. Magnusson et al. (21) reported an incidence of panoramic X-ray alterations (25%) similar to our own (24.1%) in a group of 285 patients with TMD. In 11% of the patients the authors casually identified some alteration requiring treatment. In coincidence with these investigators, we consider panoramic X-rays to be clinically useful in TMD, and we use the technique on a routine basis in these patients.

To the best of our knowledge, this is the first study on TMD involving a case-control design, based on the RDC/TMD axis I diagnostic criteria, in the Spanish and Latin American geographical setting.

## Conclusions

Signs and symptoms of TMD are common in the general population. Clenching is the parafunction establishing significant differences between TMD patients and healthy subjects considered both globally and in relation to each diagnostic category. Maximum interincisal aperture is significantly greater in the controls than in the patients, though its diagnostic usefulness is limited when used as sole indicator of TMD.
